# Welding and Riveting Hybrid Bonding of 6061 Al and Carbon Fiber Reinforced Composites

**DOI:** 10.3390/polym14010099

**Published:** 2021-12-28

**Authors:** Hongyang Wang, Bin Huang, Jinzhu Li, Nan Li, Liming Liu

**Affiliations:** Key Laboratory of Liaoning Advanced Welding and Joining Technology, School of Material Science and Engineering, Dalian University of Technology, Dalian 116024, China; hb19970820@mail.dlut.edu.cn (B.H.); 15738510261@163.com (J.L.); Linan_0819@mail.dlut.edu.cn (N.L.); liulm@dlut.edu.cn (L.L.)

**Keywords:** welding and riveting hybrid bonding, carbon fiber reinforced plastics, welding current, interface, bonding mechanism

## Abstract

Welding and riveting hybrid bonding technology was applied to join 6061 aluminum alloy and carbon fiber reinforced plastics (CFRP). The laser-arc hybrid welding process and stepped rivets were used in the experiments to reduce the impact of the poor heat resistance of composites. The effect of hybrid welding arc current on the formation and mechanical properties of 6061 Al/CFRP joints was studied. Tensile shear load up to 4.65 kN was achieved by adjusting process parameters. The welding process and mode of the fracture were analyzed. The hybrid bonded joint obtained consisted of two parts: a welded joint of Al plate and Al rivet, and a bonded interface between Al plate and CFRP plate. The mechanical properties of the hybrid joint were mainly determined by the Al plate/Al rivet welded joint. The results of the study show that there are three interfacial bonding mechanisms between aluminum and CFRP. In addition to mechanical bonding between the Al plate and CFRP plate, there were also metallurgical bonding of Al-Mg intermetallic compounds with resin matrix and chemical reactions of aluminum with resin and carbon fibers at the interface, which could improve the mechanical properties of the joints.

## 1. Introduction

Due to the severity of resource depletion and global warming, energy conservation and carbon emission reduction have become increasingly popular topics. To promote the development of a low-carbon economy, lightweight automatic equipment has attracted a large number of scientists. The most effective way to achieve the above target is to use more light alloys, such as aluminum, magnesium, and titanium [1,2,3]. As the most abundant metal element on the earth, aluminum is made into alloy by adding some other elements, such as magnesium, silicon, copper, and zinc, which is widely used in industrial production [4,5]. With the rapid development of material science, a new material named carbon fiber reinforced plastic (CFRP) was designed and used [6,7]. Since CFRP is a kind of lightweight material with more excellent mechanical properties, higher specific strength, and higher specific stiffness than steel, it plays a significant role in aerospace and automobile industries [8]. Due to the excellent performance of both aluminum alloy and CFRP, they are inevitably joined and used together. However, due to the existence of differences between the physical and chemical properties of metals and CFRP, joining them is bound to be a thorny problem [9].

At present, adhesive bonding and mechanical fastening are the two most conventional methods used to join metal and composite materials [10,11,12]. In fact, there are still a variety of shortcomings in these methods. The adhesive joint of aluminum alloy and CFRP fails easily at high temperature [13]. Mechanical properties of mechanical bonds are limited by stress concentration and mechanical damage to CFRP during the bonding process [14,15]. Besides, the weight of the joint is increased due to the weight of fasteners. Another conventional process, traditional welding, is also not suitable for the bonding of CFRP and metals. Due to the high temperature produced in the process, it is prone to causing the thermal degradation of CFRP and defects in the weld joint. In order to overcome the limitations of traditional processes, many new joining methods, especially those based on the welding process, have been investigated for joining aluminum alloy and CFRP [16,17]. Aluminum alloy and CFRP were joined successfully with laser direct joining process, and the tensile shear load of the joint reached 3000 N by adjusting the laser power and travelling speed [18]. The hybrid joints of 5052 aluminum alloy and CFRP were fabricated by friction stir spot welding. By optimizing the welding parameters, the ultimate tensile shear load reached 2690 N [19]. Besides, the ultrasonic spot welding process was also applied to join 5754 aluminum alloy and carbon fiber reinforced epoxy. The tensile shear load of the joint reached 17 MPa under appropriate parameters [20].

In addition to the innovation of welding methods, scholars have also made attempts at developing special welding structures [21,22,23]. The friction stir blind riveting (FSBR) process could be applied to join CFRP and 6111 aluminum alloy. The results showed that the spindle speed and feed rate during FSBR had little effect on the maximum lap shear tensile load of the joints, but this depends on the stack-up sequences of CFRP and aluminum alloy plates [24]. For the purpose of realizing the reliable bonding of aluminum alloy and CFRP, riveting and welding hybrid bonding, which combines the structure of riveting with the method of welding, was applied in this study.

Key to the problem of joining Al and CFRP dissimilar materials by various welding methods is control over the heat input so as to reduce the thermal degradation of CFRP [25]. The weld by laser-arc hybrid welding process is narrow and deep, and the width of the heat affected zone can be reduced effectively [26]. Therefore, the combination of this hybrid welding technology and riveting process can be considered an effective way to join Al alloy and CFRP. A previous study obtained well-formed and strong joints of 6061 aluminum and carbon particle reinforced polyether-ether-ketone (CPRP) with laser-TIG (tungsten inert gas) welding assisted riveting hybrid joining technology. Besides, its bonding mechanism consisted of three parts: the welded joint of the aluminum alloy plate and rivet, the mechanical interlocking function of the rivet, and the mechanical bonding and chemical reaction on the Al/CPRP interface [22].

The forming quality and mechanical properties of the aluminum alloy welded joint are sensitive to the change of welding current. The half width of weld increases with increasing welding current, whereas the weld pool depth is relatively unchanged [27]. Besides, the high temperature attained in the molten pool can result in the thermal degradation of CFRP, which is harmful to weld formation and quality. Therefore, the control of heat source is of significant importance to ensure the quality of Al/CFRP hybrid joints.

Based on previous studies, the effect of welding current on the mechanical properties and bonding methods of the Al/CFRP joints was researched in this paper. The target is to control the thermal degradation of CFRP and the formation of welds, which is the prerequisite for obtaining Al/CFRP joints with excellent mechanical properties. The tensile shear load of the joints was tested to explore the influence of welding current on the mechanical properties of Al/CFRP hybrid bonding joints. Moreover, for the purpose of figuring out the bonding methods of the Al/CFRP joints under appropriate process parameters, the microstructure and elemental distribution at the interface were analyzed by scanning electron microscopy (SEM), electron probe microanalysis (EPMA), and X-ray photoelectron spectroscopy (XPS).

## 2. Materials and Methods

**Figure 1:**
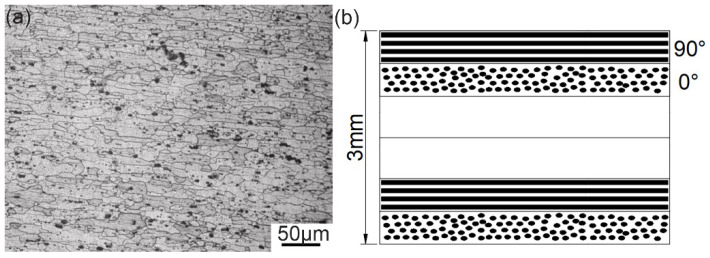
Microstructure of (**a**) 6061 Al and (**b**) CF-PEEK.

Carbon fiber reinforced polyether ether ketone composite (CF-PEEK) plates with the size of 100 mm × 25 mm × 3 mm purchased from Junhua Ltd. (Changzhou, China) and 6061 aluminum alloy plates with the size of 100 mm × 25 mm × 1.5 mm were used in the experiments. The microstructure of 6061 aluminum alloy and the distribution of carbon fiber in CF-PEEK are shown in Figure 1. It can be observed that the carbon fiber fabric was a layered structure, and the orientation of carbon fibers was [90/0°]. The physical properties of 6061 aluminum alloy and CF-PEEK were quite different, as shown in Table 1.

**Figure 2:**
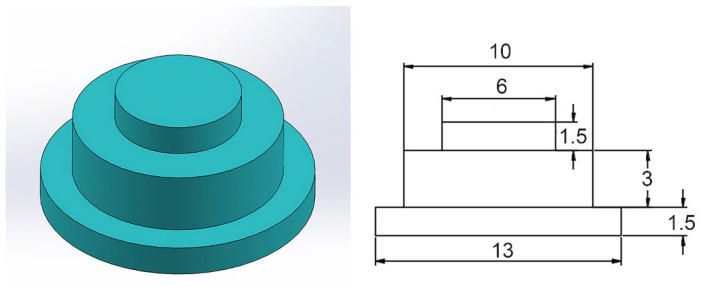
Schematic of Al rivet.

For the purpose of decreasing the thermal damage and decomposition of carbon fiber reinforced plastics (CFRP), the rivets used in the experiment were processed into the dimensions shown in the Figure 2. The dimensions of the three steps were ϕ13 mm × 1.5 mm, ϕ10 mm × 3 mm and ϕ6 mm × 1.5 mm, respectively. The size of the lapping area was 30 mm × 25 mm. In order to assemble the two plates and rivets, CFRP plates and Al plates were drilled with holes of ϕ10 and ϕ6 mm in the center of the lap areas, respectively. Before assembling, the surfaces of the plates were polished with sandpaper and then cleaned with anhydrous ethanol.

**Figure 3:**
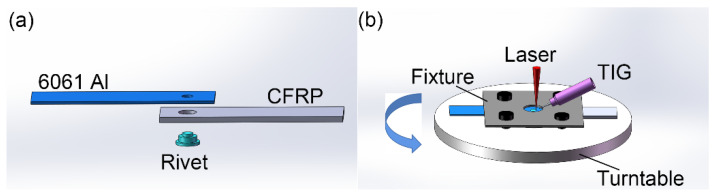
(**a**) The schematic diagram of assembling and (**b**) laser-TIG hybrid welding process.

The processes of welding and riveting hybrid bonding are shown in Figure 3. Firstly, the aluminum alloy rivet passed through the CFRP plate and aluminum plate in turn. Next, the sample was clamped on the rotary table by a fixture, with aluminum plate on the top and CF-PEEK plate on the bottom, as shown in Figure 3a. In order to achieve the effective joining between aluminum alloy and CF-PEEK, two materials with great differences in physical and chemical properties, a laser-arc hybrid heat source was applied in the welding process. The laser irradiated the circumference along the direction perpendicular to the aluminum plate at an angle of 45° with arc. The horizontal distance between laser beam and tungsten electrode was 1.5 mm and the tungsten electrode was mounted at a height of 1.5 mm on the upper surface of the aluminum plate. During the welding process, the laser-arc hybrid heat source was fixed while the specimen rotated around it. The laser and welding arc were controlled by a preset program. The laser and welding currents were immediately switched off automatically after the specimen rotated for one circle so as to obtain a complete circular welded joint.

**Figure 4:**
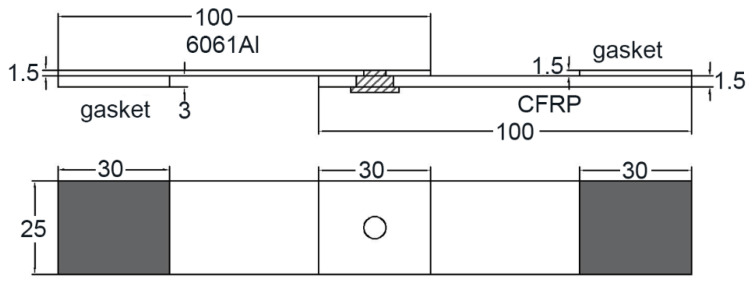
The diagram of the tensile shear specimen.

A low-power pulsed laser with rectangular waveform was used in the experiment, whose maximum rated power was 800 W. An OTC AEP-500P AC/DC was used as the arc welding machine, which could output stable AC/DC from 0 to 500 A. The TIG (tungsten inert gas) current varied from 70 A to 110 A (70, 80, 90, 100, and 110 A) and the laser with an average power of 400 W were used in this study. The laser defocusing amount was 0 mm. The welding speed was 540 mm/min, and the corresponding rotary speed of the specimen was 28.6 r/min. The tensile shear load of the specimens was tested by an electronic universal testing machine at the speed of 2 mm/s. The diagram of the tensile shear specimen is shown in Figure 4.

The microstructure of cross sections of hybrid bonded joints was observed by SEM (ZEISS Group, Oberkochen, Germany) and EPMA (JEOL Ltd., Tokyo, Japan). The tensile shear fracture was observed by SEM in order to figure out the failure mode of the joint. EPMA was applied to analyze the elemental distribution at the interface between aluminum alloy and CFRP, which was observed by face-scanning analysis and energy-dispersive spectroscopy (EDS). Moreover, element valence at the interface was used to analyze XPS (Thermo Fisher Scientific Inc., Waltham, MA, USA) data to confirm the chemical reaction of aluminum alloy and composite materials.

## 3. Results and Discussion

### 3.1. The Morphology and Microstructure of Al/CFRP Hybrid Bonded Joints

**Figure 5:**
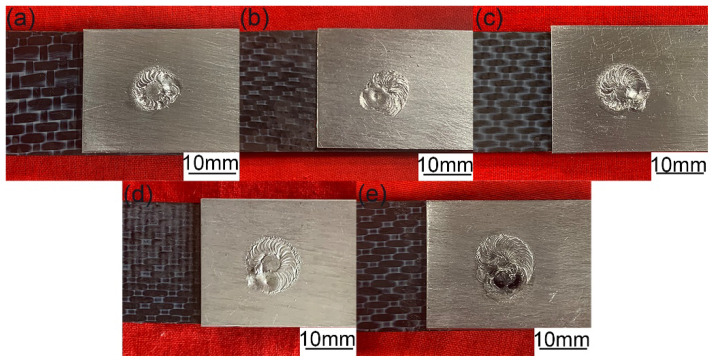
The top appearances of hybrid bonding joints under different welding currents: (**a**) 70 A, (**b**) 80 A, (**c**) 90 A, (**d**) 100 A and (**e**) 110 A. (Plaser = 400 W, Welding speed = 540 mm/min).

The laser-arc hybrid welding source was used in the welding and riveting hybrid bonding of Al to CFRP process. In the stepped structure welding and riveting hybrid bonding joint, the welding width of the stepped part had obvious effects on the properties of the joints. In the laser arc hybrid welding process, the welding arc current became the key parameter that affected the width of the molten pool. Therefore, the influence of the welding arc on the welding process and properties of the hybrid bonded joints was discussed in this study. The top appearances of hybrid bonded joints obtained by different welding currents are shown in Figure 5. Judging from the appearances, the welded joints were well formed under the welding currents from 70 to 100 A. However, when the welding current was raised to 110 A, obvious defects and black substances appeared in the welded joint, which can be attributed to the decomposition of the CFRP.

**Figure 6:**
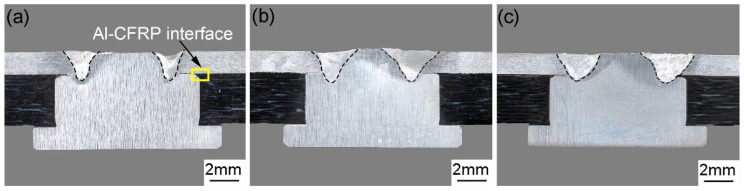
Cross-sectional macrostructures of Al/CFRP bonding joints obtained by different welding currents: (**a**) 70 A, (**b**) 90 A and (**c**) 100 A.

Figure 6 presents the cross-sectional macrostructure of welding and riveting hybrid bonded joints of Al and CFRP. The Al plate-Al rivet welded joint was roughly funnel-shaped under the action of the laser-arc hybrid welding source. The aluminum plate and the third step of the rivet were completely melted and bonded with the partially melted second step. As shown in Figure 6, there are no obvious defects in the welded joint, and the width of weld is increased obviously with the increasing welding currents.

**Figure 7:**
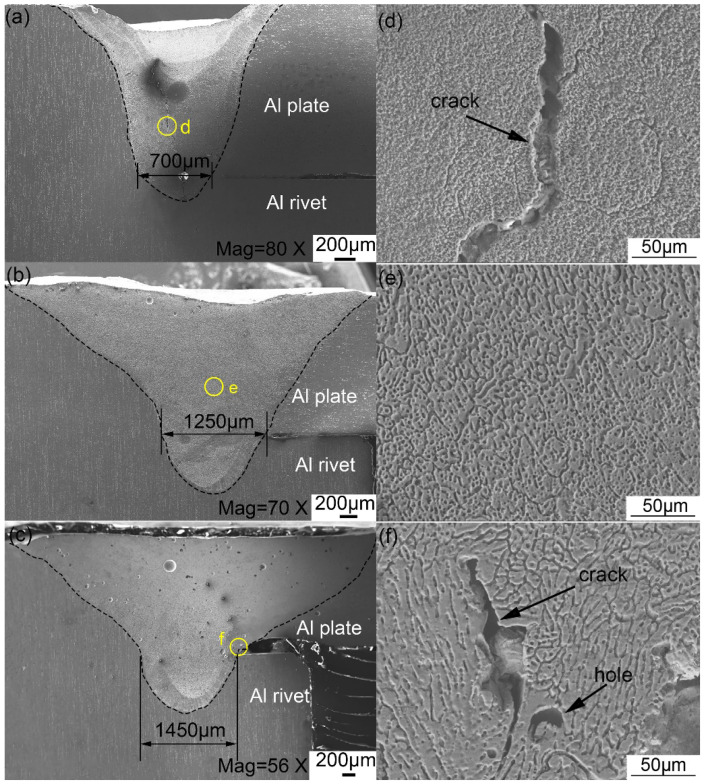
The cross section of welded joints under different welding currents: (**a**) 70 A, (**b**) 90 A and (**c**) 100 A; Microstructure of the welded joints: (**d**) 70 A, (**e**) 90 A and (**f**) 100 A.

Welding current makes an important impact on the welding and riveting hybrid bonded joint of Al alloy and CFRP, which is proven by the experimental results shown in Figure 7. In this study, the power of laser beam was constant so that there was no significant change in the depth of the hybrid joints under different welding currents. In the case of the laser beam power of 480 W, the width of molten metal at the second step of the rivet increased with the welding current. When the arc current was 70 A, the welding width of the fusion zone was about 700 μm. However, there are some cracks at the center of the welded joint obtained by a welding current of 70 A, which can be seen from the cross section shown in Figure 7a. The cracks were caused by the incomplete penetration of aluminum plate due to insufficient heat input. As a consequence, the strength of this joint was relatively weak. When the welding arc current was 90 A, the width of the joint at the second step of the rivet was about 1250 μm, which was much wider than that of 70 A. Besides, no obvious cracks or holes in the weld can be found in Figure 7b,e. With the increase of welding arc currents, the heat input from the hybrid welding source increased. When the welding current increases to 100 A, the width of the fusion zone increases to 1450 μm, and there were a number of pores and cracks in the weld, as shown in Figure 7c,f. The high temperature generated by the laser and arc hybrid welding source caused the thermal degradation of the composite material. Thus, the gas produced by the process impacted the molten metal and led to obvious defects, such as pores and cracks in the fusion zone of aluminum plates and the second step of the rivet. Therefore, the strength of the joint was not as high as that of 90 A.

### 3.2. Mechanical Properties

**Figure 8:**
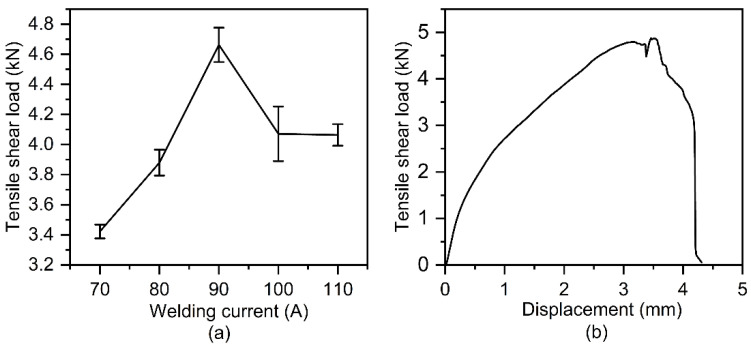
(**a**) The tensile shear load of joints under different welding currents. (**b**) Tensile shear curves of the 90 A joint.

The tensile shear load of the joints obtained by the hybrid bonding method were tested in order to evaluate their strength. Figure 8a illustrates the tensile shear peak load of the welded and riveted hybrid joint between 6061 Al and CFRP fabricated by different welding currents. Under 90 A, the tensile shear load increased with increasing welding current. When the welding current reached 90 A, the peak load of the joint was about 4.65 kN. Over 90 A, the tensile shear load tended to decrease with the increase of welding current. When the welding current increased to 100 A, as well as 110 A, the peak load of the joints was reduced to about 4.1 kN.

**Figure 9:**
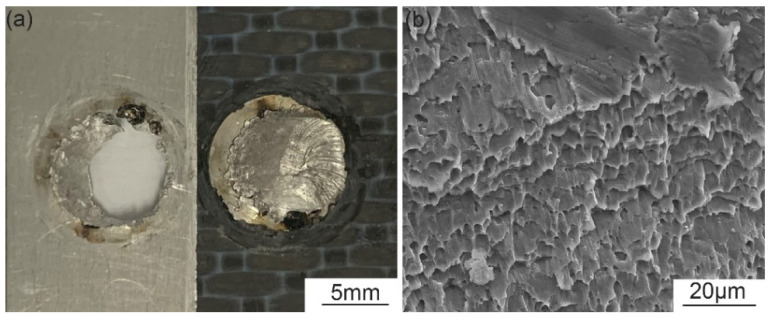
(**a**) Macrostructure and (**b**) microstructure of fracture.

**Figure 10:**
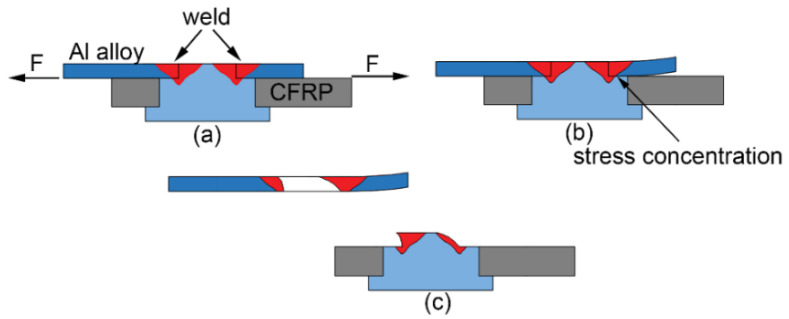
The diagram of the fracture process of the tensile shear test: (**a**) start testing; (**b**) deformation and (**c**) fracture.

The failure modes of tensile shear test samples of welded and riveted hybrid joints were all rivet pull-out fracture, which meant that the rivets were pulled out from the aluminum alloy plate along the circular weld area. In Figure 9a, obvious bending deformation appears at the top of the rivet. Figure 9b shows the microstructure of the fracture. As shown in Figure 8b, after the tensile load reaches the peak value, the joint still had a certain amount of deformation, rather than being immediately fractured, which means that the joint has better plasticity. Figure 10 depicts the fracture process of the tensile shear test. When the load increased to a certain value, due to the differences between the thickness and strength of the aluminum plate and the composite plate, the aluminum plate approaching the CFRP plate warped first. This led to stress concentration in the welded joint near the CFRP plate. With an increase of tensile shear load, the top step of the rivet gradually slid, before the crack gradually expanded along the weld from the stress concentration position, and finally completely separated.

### 3.3. Bonding Mechanisms of Al/CFRP Interface

**Figure 11:**
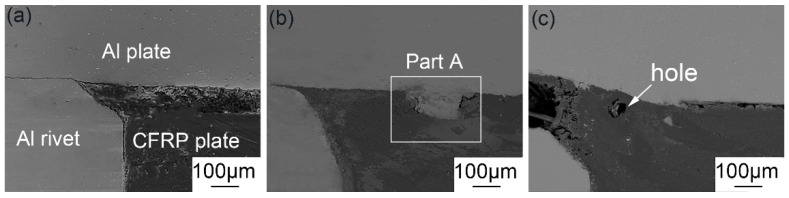
The morphologies of the interface between 6061 Al and CFRP with different currents: (**a**) 70 A, (**b**) 90 A and (**c**) 100 A.

The bonding mechanisms of the interface between 6061 aluminum alloy and CFRP were indicated by the microstructure and electron probe (EPMA) results of the joints. Figure 11 corresponds to the Al/CFRP interface area marked in Figure 6a. As can be seen in Figure 11b, there is partially melted aluminum alloy visibly embedded in the CFRP plate at the interface of the 90 A joint. With the welding current increased to 100 A, the molten metal enlarged with higher temperature. At this higher temperature, the resin near the interface was violently degraded, resulting in the generation of the large hole in CFRP, as shown in Figure 11c. The generated gas caused the resin in a molten state to flow at a high temperature, causing the bottom of the aluminum to sink inward. In addition, the gas led to holes and cracks in the welded joint of the Al plate and Al rivet, as shown in Figure 7f. Therefore, the strength of 100 A welded joint was significantly lower than that of the 90 A joint.

**Figure 12:**
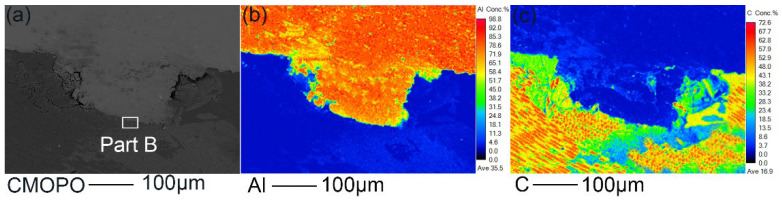
(**a**) Microstructure of Al plate/CFRP plate interface. Face-scanning results of (**b**) Al and (**c**) C.

Under sufficient heat input, the molten aluminum can form an obvious bonding with the CFRP plate at the interface, and this bonding is most effective when the welding arc current is 90 A, as shown in Figure 11. Therefore, the 90 A joint was selected for subsequent microscopic analysis. In order to further understand the bonding structure of the joint under the action of the hybrid heat source, EPMA was used to analyze the interface between Al and CFRP. EPMA results of part A at the Al plate/CFRP plate interface in Figure 11b are shown in Figure 12. It can be seen from the distribution of Al element that serrated molten metal about 150 μm high penetrated through the first layer of 90° fibers and then embedded into the second layer of 0° fibers, which meant a tight bonding formed between Al plate and CFRP. At the same time, the melted resin adhered to the surface of the aluminum alloy, improving the strength of the bonding. It can be judged from the melting of aluminum that the temperature at the interface was close to the melting point of 6061 aluminum alloy, which was higher than the thermal degradation temperature of polyether ether ketone composite (PEEK) resin at 570 °C [19,28]. In addition, the carbon fibers that originally existed in the position of serrated aluminum alloy particles underwent a chemical reaction. The gas produced by the thermal degradation of CFRP led to gaps between the aluminum alloy and CFRP at the interface. Since the gas generated was not enough to influence the welding process, the welded joint was well formed. Due to the low thermal conductivity of carbon fiber reinforced polyether ether ketone composites (CF-PEEK) shown in Table 1, only a small part of the CFRP at the interface was thermally degraded. Therefore, the strength of the joint would not be significantly affected by the slight thermal degradation of PEEK resin. Based on the above phenomenon, it can be confirmed that effective mechanical bonding was formed between the aluminum plate and CFRP plate, though there were minor defects at the interface.

**Figure 13:**
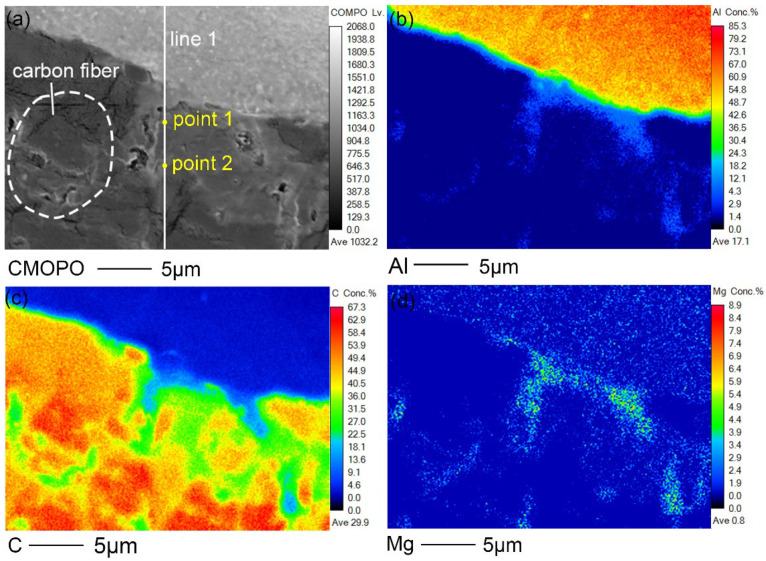
(**a**) Microstructure of part B at the interface, face scanning results of (**b**) Al, (**c**) C and (**d**) Mg.

**Figure 14:**
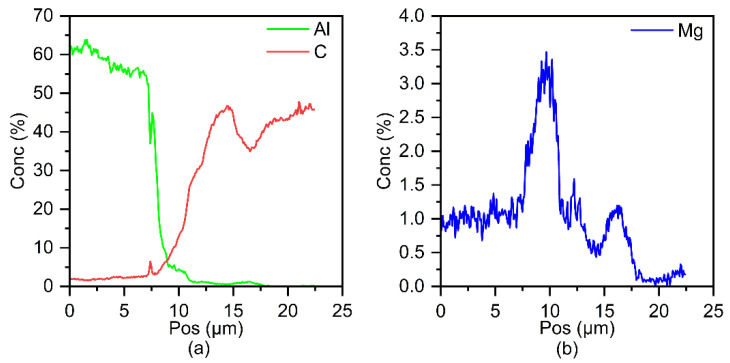
Line scanning results of (**a**) Al, C and (**b**) Mg on line1 in Figure 13a.

Figure 13a presents the high-magnification microstructure of part B in Figure 12a. It can be observed that the aluminum alloy in this area formed an excellent fusion with CFRP. Inside the dashed circle was a complete carbon fiber whose diameter was about 5 μm and the carbon fibers nearest to the Al/CFRP interface reacted chemically with the aluminum alloy. To further reveal the bonding mechanism of the Al/CFRP interface, surface scanning was performed by EPMA to obtain the distribution of Al, C, and Mg elements, as shown in Figure 13b–d. Moreover, in order to further understand the distribution of main elements at the Al/CFRP interface, line scanning was performed on the position shown by line 1. The results are shown in Figure 14. The distribution of carbon indicates that some carbon fibers at the interface were incomplete, which may also have a chemical reaction at high temperature. At the interface, the Al content decreased gradually along the side of CFRP plate, while the content of C decreased gradually along the side of aluminum plate. It can be inferred from face-scanning results shown in Figure 13b,c and the line scan results of Al and C shown in Figure 14 that Al and C elements formed a transition layer with a width of about 5 μm at the interface.

According to Figure 13b,d, Al and Mg elements diffused into CFRP along river like channels and were dispersedly distributed in it. In general, however, Al is difficult to diffuse in C in the form of a simple substance. The contents of Al and Mg elements at point 1 in Figure 13a are 3.81 at% and 2.81 at% respectively. Besides, the contents of Al and Mg elements at point 2 are 0.66 at% and 0.47 at% respectively. Significantly, Mg element was enriched at the interface. The results suggest that Al and Mg elements diffused in CFRP in the form of Mg-Al intermetallic compounds. It can be judged from Figure 13b,d and Figure 14 that the Mg-Al intermetallic compounds have penetrated into the interior of the resin matrix more than 5 μm away from the interface. This is the key evidence of metallurgical bonding between 6061 aluminum alloy and CFRP.

**Figure 15:**
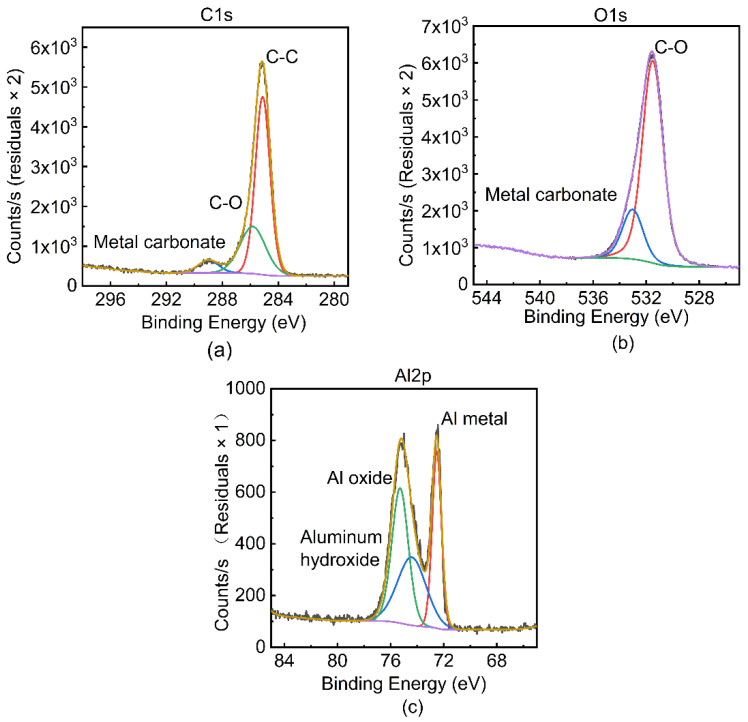
XPS results of: (**a**) C, (**b**) O and (**c**) Al at the Al/CFRP interface.

For the purpose of confirming the existence of a chemical reaction between the aluminum and PEEK resin, XPS was applied to analyze valence and bonding states of main elements. In order to avoid the influence of the oxide film on the experimental results, argon ion etching was used to thin the sample by 30 nm. The XPS analysis results of C, Al, and O are shown in Figure 15. The spectra of Al show that, in addition to aluminum metal and aluminosilicate, aluminum oxide was also measured at the interface after argon ion etching for 90 s, which can also be proven by the spectrums of O. Meanwhile, the existence of aluminum carbonate can be judged by the spectrums of C. Based on the above results, it can be concluded that part of the composite material near the interface was thermally degraded under the action of the high temperature transferred from molten aluminum, generating oxygen, carbon dioxide, and organic gases at the Al/CFRP interface, which reacted with molten aluminum and formed aluminum oxide and a small amount of metal carbonate. The detected aluminum hydroxide was probably formed by the hydrolysis of aluminum carbonate. In summary, it is evident that chemical reactions occurred at the interface between aluminum alloy and CFRP during the welding process.

Based on the above experimental results, in addition to the micromechanical connection formed by serrated molten aluminum alloy and CFRP, there was also a chemical interaction between them. These two bonding mechanisms work together at the Al/CFRP interface to further improve the strength of the joint.

## 4. Conclusions

Welding and riveting hybrid bonding was used to join 6061 aluminum alloy and carbon fiber reinforced plastics (CFRP). The mechanical properties and bonding modes of the joints were discussed. The followings are the conclusions.

(1) The mechanical properties of the hybrid joints were mainly determined by the welded joints. The welding current significantly affected the mechanical properties of the joints. The tensile shear load increased firstly, then decreased with increasing welding current, and reached the maximum (4.65 kN) at 90 A. In the case of 90 A, the width of molten aluminum at the second step of the rivet was about 1250 μm without obvious defects in the weld. However, a further increase of the welding current would intensify the decomposition of the CFRP, which could have an obviously harmful effect on the formation and mechanical properties of the joint.
(2) The hybrid bonded joint obtained by appropriate parameters can be divided into two parts: Al plate/Al rivet welded joint and Al plate/CFRP plate bonding joint at the Al/CFRP interface. In addition, the bonding mechanism of the Al/CFRP interface consisted of micromechanical bonding, metallurgical bonding, and chemical bonding. The combined effect of the above bonding modes was the source of the strength of the hybrid bonded joint.

## Figures and Tables

**Table 1:** The physical and mechanical properties of 6061 aluminum alloy and CF-PEEK.

Material	Melting Point (°C)	Density (g·cm^−3^)	Thermal Conductivity (W·m^−1^·K^−1^)	Tensile Strength (MPa)
6061 Al	580–650	2.70	167	320
CF-PEEK	330	1.59	2.0	689

## Data Availability

Data is contained within the article.
